# The association of high-sensitivity c-reactive protein and other biomarkers with cardiovascular disease in patients treated for HIV: a nested case–control study

**DOI:** 10.1186/1471-2334-13-414

**Published:** 2013-09-03

**Authors:** Andrea De Luca, Katleen de Gaetano Donati, Manuela Colafigli, Alessandro Cozzi-Lepri, Amalia De Curtis, Andrea Gori, Laura Sighinolfi, Andrea Giacometti, Maria Rosaria Capobianchi, Alessandro D’Avino, Licia Iacoviello, Roberto Cauda, Antonella D’Arminio Monforte

**Affiliations:** 1 University Division of Infectious Diseases, University Hospital of Siena, Siena, Italy; 2 Institute of Clinical Infectious Diseases, Catholic University, Rome, Italy; 3 Research Department of Infection and Population Health, University College London, London, UK; 4 Department of Epidemiology and Prevention, IRCCS Istituto Neurologico Mediterraneo Nueromed, Laboratory of Molecular and Nutritional Epidemiology, Pozzilli (IS), Italy; 5 Department of Infectious Diseases, Hospital of Monza, Monza, Italy; 6 Department of Infectious Diseases, Hospital of Ferrara, Ferrara, Italy; 7 Department of Infectious Diseases, Marche Polytechnic University, Ancona, Italy; 8 Department of Virology, IRCCS Ospedale Lazzaro Spallanzani, Rome, Italy; 9 Institute of Infectious and Tropical Medicine, University of Milan, Milan, Italy; 10 Istituto di Clinica delle Malattie Infettive, Università Cattolica del Sacro Cuore, Largo F Vito 1, 00168, Roma, Italy

**Keywords:** Biomarkers, Cardiovascular disease, HIV, hsCRP

## Abstract

**Background:**

Elevated high-sensitivity C-reactive protein (hsCRP) increases the risk of cardiovascular disease (CVD) in the general population, but its role as a predictive marker in HIV-positive patients remains unclear. Aim of the study was to evaluate whether hsCRP or other biomarkers are independent predictors of CVD risk in HIV-infected patients.

**Methods:**

Retrospective, nested case–control study. HIV-positive men and women (35–69 years of age) receiving combination antiretroviral therapy (cART) were included. Cases (n = 35) had a major CVD event. Controls (n = 74) free from CVD events for at least 5 years from starting ART were matched on diabetes and smoking. HsCRP, D-dimer, P-selectin, interleukin-6 (IL-6), tissue plasminogen activator, plasminogen activator inhibitor-1 levels were measured.

**Results:**

High hsCRP was associated with CVD risk, independently of traditional cardiovascular risk factors, HIV replication and the type of ART received at the time of sampling (adjusted odds ratio 8.00 [1.23-51.94] comparing >3.3 mg/L with <0.9 mg/L; *P* = 0.03). Higher IL-6 and P-selectin levels were also independently associated with increased CVD risk, although the association was weaker than for hsCRP. Higher total cholesterol and lower HDL cholesterol increased CVD risk, independent of hsCRP.

**Conclusion:**

hsCRP may be a useful additional biomarker to predict CVD risk in HIV-infected patients receiving cART.

## Background

Combination antiretroviral therapy (cART) has substantially reduced HIV-related morbidity and mortality [1]. As a result, HIV-infected people have longer life-expectancy and the focus of therapy has shifted to long-term management of the infection, particularly treatment-related side-effects and comorbidities. Cardiovascular disease (CVD) has received particular attention, as it is associated with considerable morbidity and mortality [2], and there is cumulating data showing that HIV-infected patients are at higher risk of CVD than uninfected controls [3,4]. However, controversy exists over the relative contribution of host factors (e.g. smoking, hypertension, diabetes, male sex and older age), rather than the virus itself or use of cART to this increased risk. For HIV infection and cART, this effect may occur via their modification of traditional risk factors [5], as well as pathogenic pathways leading to atherosclerosis and CVD (e.g. inflammation and endothelial dysfunction) [3,6-10].

Although several studies have suggested a positive association between cART and CVD risk [3,11,12], there is also evidence that cART may improve endothelial function and protect against atherosclerosis [13,14]. Furthermore, differences exist in the relative risk of CVD between, and within, antiretroviral drug classes [15-21]. In particular, the Data Collection on Adverse Events of Anti-HIV Drugs (D:A:D) Study Group found an association between the use of specific protease inhibitors, of abacavir and of didanosine and an increased risk of myocardial infarction [22], but not all subsequent studies have confirmed this result [23].

Research into CVD risk factors in the general population has identified a number of predictive biomarkers, such as apolipoprotein B, total cholesterol, interleukin-6 (IL-6), serum amyloid A, D-dimer, tissue plasminogen activator (t-PA), plasminogen activator inhibitor-1 (PAI-1), P-selectin, intercellular adhesion molecule 1 (ICAM-1), vascular adhesion molecule 1 (VCAM-1) and, most importantly, C-reactive protein (CRP) as determined by high sensitivity techniques (hsCRP) [24-29]. HsCRP levels <1.0 mg/L, 1.0–3.0 mg/L and >3.0 mg/L indicate low, average and high CVD risk, respectively [30].

Given the increased risk of CVD in HIV-infected patients, the predictive role of such biomarkers in this patient population is of interest. Several potential CVD markers have been investigated in HIV-positive patients, including lipoprotein particles, measurements of carotid artery intima–media thickness and of arterial stiffness, tumor necrosis factor-α, IL-6, IL-10, myeloperoxidase, platelet (P)-selectin, leptin, D-dimer, adiponectin, soluble VCAM-1, ICAM-1 and ICAM-3, and hsCRP [31-33]. A recent analysis on the SMART study found an association between CRP, IL-6 and d-dimer and the risk of CVD events [34].

Our primary objective was to evaluate whether hsCRP predicts CVD risk in HIV-infected patients, independently of other established risk factors, using the data of persons enrolled in two Italian cohorts. Our secondary objective was to investigate the association between other biomarkers and CVD.

## Methods

### Participants

We studied patients enrolled in two cohort studies; the Icona Foundation Cohort and the Catholic University of the Sacred Heart (CUSH) clinical database, Rome. In CUSH, data have been collected as part of routine clinical care since 1997 (full details are reported elsewhere [35]). The Icona Foundation Study includes HIV-positive patients presenting at 67 infectious disease centers across Italy and represents the continuation of the Italian Cohort of Antiretroviral-Naïve Patients (I.Co.N.A) study, which has previously been described [36]. After enrolment, follow-up clinical visits occur approximately every 6 months in the Icona Foundation Study and every 3 months in CUSH.

All patients provided written consent to participate in Icona Foundation Study and in CUSH, following procedures in accordance with the ethical standards of the responsible committee on human experimentation and the Helsinki Declaration. In both studies, patients informed consent includes a specification for use of samples for further research in HIV. No specific consent for inclusion in the current analysis was therefore needed. The studies and the corresponding informed consents were approved by the Ethics Committees of the various ICONA clinical sites (see Appendix) and that of the Catholic University for CUSH. Inclusion criteria for this analysis were age 35–69 years and no history of a major CVD event before starting cART and no evidence of any other inflammatory disorder over the 3 months prior to the date of the stored plasma sample. Exclusion criteria were current use of active hormonal-based therapy and of therapy with anti-inflammatory drugs or statins. A small proportion of patients had used these drugs prior to the date of the stored sanple.

### Analysis design

We conducted a retrospective, matched case–control study nested within CUSH and the Icona Foundation Study involving HIV-infected patients with CVD (cases) or without CVD (controls). CVD cases were defined as HIV-infected patients who had a major cardiovascular event (acute myocardial infarction; stable or unstable angina or were undergoing myocardial revascularization procedures) while receiving cART (a regimen including at least 3 antiretrovirals), and for whom at least one plasma sample was stored before the CVD event. Within each cohort, HIV-infected controls (a minimum of two per each case) were defined as patients who were currently free of CVD events (i.e. had never developed CVD events), had at least 5 years of follow-up after cART initiation and had at least one plasma sample stored over the observation period.

Cases and controls were matched on previous history of diabetes and smoking status. The ‘index time’ for analysis was fixed at the date of the CVD event for cases and at last follow-up for controlsBecause date of sample for controls was on average much more recent than that of cases, calendar year and time between the date of sample and the index time were included as an additional adjusting factor in the multivariable analyses.

### Sample collection

In CUSH, plasma samples are collected each time a viral load assay is performed (i.e. approximately every 3 months), and stored at −80°C. In the Icona Foundation Study, plasma samples are stored at the time of enrolment and at least once a year. In both cohorts, plasma samples are collected using Vacutainer® collection tubes (lavender top, containing ethylenediaminetetraacetic acid, Becton Dickinson, New Jersey, USA). In the case of ICONA foundation Study, samples are initially kept frozen at the various clinical sites at −20°C or −80°C and subsequently shipped using dry ice to a central repository in Rome, Italy, where they are kept at −80°C until use for retrospective analysis. Dates of sampling were (ranges) 1999–2008 for cases and 2006–2008 for controls for CUSH, and 1998–2004 for cases and 2000–2004 for controls for the Icona Foundation Study. As a general rule, the most recent plasma sample stored before the analysis index time was analyzed in both cases and matching pair of controls (late samples). However, for some cases and controls, if more than one stored sample was available, biomarkers were measured using an additional earlier sample (early samples). To avoid bias, laboratory personnel was blind to whether the sample was coming from a case or a control. Samples, which were labelled with a unique identifier so that they could be linked with patients ID and their clinical data for statistical analysis, were analyzed in random order.

### Biochemical analyses

Total cholesterol and HDL were measured using standard techniques by local laboratories, while biomarkers were analyzed by the Laboratory of Genetic and Environmental Epidemiology, Catholic University of Campobasso, Italy. hsCRP was measured by nephelometry using an automatic analyzer (ILab 350, Instrumentation Laboratory, Milan, Italy). Quality control was performed using an internal laboratory standard; the inter-day coefficient of variation (CV) was 2%. D-Dimer was evaluated utilizing a latex particle-enhanced immunoturbidimetric assay (IL ACL9000, Instrumentation Laboratory, Milan, Italy). Quality control was performed using an in-house citrate plasma pool; inter-and intra-day CV was 7.6% and 5.4%, respectively. P-selectin (Bender MedSystems Europe, Vienna, Austria), IL-6 (R&D System Europe, Abingdon, UK), t-PA and PAI-1 (American Diagnostica Inc. GmbH, Pfungstadt, Germany) were measured utilizing commercially available enzyme-linked immunosorbent assay (ELISA) kits, as specified by the manufacturers. The intra-assay CV, using in-house citrate plasma, were, according to the manufacturer, 4.3%, 10.5%, 4.8% and 9.8% respectively.

The detection limit of the different assays were 1.06 ng/ml for P-selectin, 1.0 ng/ml for t-PA, 1.0 ng/ml for PAI-1 and 0.70 pg/ml for IL-6.

### Sample size calculation and statistical analysis

By setting the type I error α at 5%, and assuming that 40% of patients had a hsCRP level above 3 mg/L [30] and two controls per case, we calculated that 35 cases and 70 controls were necessary to achieve 80% power to detect an OR for CVD risk of 3.3 for a hsCRP level above the pre-defined threshold (vs. below 1 mg/L). Smaller effects were expected for the other biomarkers which the study was not powered on.

Summary statistics and illustrations appropriate for matched case–control studies were used to summarize key findings [37]. Conditional logistic regression analysis for matched case–control studies was performed to evaluate the association between the baseline characteristics of the patients as well as the levels of each biomarker with the risk of CVD events. Separate analyses were performed using the late samples as well as using all samples combinedby tertiles of each biomarker (for the early samples, sparse data led to numerical problems in the multivariable analysis, resulting in an unstable model); cut-offs for the tertiles were chosen using the distribution of the measures in the combined dataset. For hsCRP, sensitivity analyses were also performed using the categories of <1, 1–3 and >3 mg/L as these had been clinically validated in the general population.

Models with log_2_ transformed biomarker levels were also used, estimating the increased risk of CVD associated with a one log_2_ difference in biomarker levels. Separate models were fit for each biomarker and, because of the large number of parameters involved, we decided *a priori* not to fit the model mutually controlling for all biomarkers. The following covariates assessed at the date of stored samples were considered for the adjusted analyses: calendar year, duration of time between the date of sample and the analysis time, age, total cholesterol, HDL, CD4^+^ cell count and viral load, cumulative exposure to nonnucleoside reverse transcriptase inhibitors (NNRTIs), nucleoside reverse transcriptase inhibitor (NRTIs) and protease inhibitor (PIs) prior to sample, and co-infection with hepatitis B or C. [38]. Sensitivity analyses, were performed on the combined samples set additionally controlling for one of these factors at the time: body mass index (BMI), estimated glomerular filtration rate (eGFR, by the MDRD formula) and prior use of statins. In the analysis using the combined data set, standard errors were adjusted for non-independence between biomarkers coming from the same individual using the cluster option for *clogit* in STATA [39]. All statistical analyses were performed using SAS version 9.1, Cary, North Carolina, United States and STATA software (StataCorp. 2008. Stata Statistical Software: Version 10.1, College Station, Texas, USA). All tests were two-sided and assumed a level of significance of 0.05.

## Results

### Baseline characteristics

We studied 109 patients (35 cases, 74 controls) of whom 17 cases, 40 controls were from CUSH and 18 cases, 34 controls from the Icona Foundation Study. The distribution of the matching variables in cases and controls was: smokers/diabetics (3;4), non-smokers/diabetics (4;8), smokers/non-diabetics (22;50), non-smokers/non-diabetics (6;12). Characteristics of cases and controls at the time of the late samples are summarized in Table 1. Compared with controls, cases had higher total cholesterol, a shorter cumulative exposure to ART, regardless of drug class and alate sample that was stored less recently, but closer to the analysis time (Table 1). The index pathology for the 35 cases was: acute myocardial infarction (n=30), revascularization procedures (n=1), stable or unstable angina (n=4).

**Table 1:** Main characteristics of cases and matched controls at time of late sample

***Characteristic***	**Cases**	**Controls**	**p-value#**
	(n= 33)*	(n= 71)*	
Age, years			
median (IQR)	47 (42, 53)	45 (39, 49)	0.139
Gender, n(%)			
Female	1 (3%)	8 (11%)	0.205
Mode of HIV trasmission, n (%)			
IDU	13 (39%)	21 (30%)	
Homosexual contacts	7 (21%)	18 (25%)	0.365
Heterosexual contacts	12 (36%)	24 (34%)	0.657
Other/Unknown	1 (3%)	8 (11%)	0.143
Co-infection with HBV or HCV, n (%)			
Yes	16 (48%)	26 (37%)	0.224
CD4 count, cells/mm^3^			
Median (IQR)	550 (451, 730)	525 (370, 768)	0.805
HIV RNA, log_10_ copies/mL			
Median (IQR)	2.1 (1.7, 4.0)	1.7 (1.7, 2.7)	0.164
Median total cholesterol (IQR)			0.006
nmol/L	5.4 (4.6, 6.3)	4.9 (4.0, 5.6)	
mg/dL	209.0 (180.0, 247.0)	192.0 (155.0, 217.0)	
Median HDL-cholesterol (IQR)			0.130
nmol/L	2.1 (1.8, 2.3)	2.3 (1.9, 2.6)	
mg/dL	40.0 (35.0, 45.0)	44.0 (37.0, 51.0)	
cART-treated before late sample, n (%)			0.115
Yes	29 (88%)	54 (76%)	
AIDS diagnosis, n (%)			0.586
Yes	6 (18%)	16 (23%)	
Cumulative exposure to NRTI, months			0.003
Median (IQR)	51 (27, 102)	121 (40, 145)	
Cumulative exposure to NNRTI, months			0.021
Median (IQR)	3 (0, 27)	30 (0, 77)	
Cumulative exposure to PI, months			0.020
Median (IQR)	26 (8, 38)	52 (11, 89)	
Calendar year of sample			<.001
Median (IQR)	2003 (2001, 2006)	2008 (2003, 2008)	
Median duration between late sample and analysis time, months	3 (2, 12)	8 (7, 28)	0.051
Prior use of statins°, n (%)			0.263
Yes	5 (15%)	4 (6%)	
Body Mass Index§, kg/m^2^			0.366
Median (IQR)	23.7 (21.7, 25.8)	23.1 (21.3, 26.3)	
eGFR^ (MDRD formula), ml/min/1.73 m^2^			0.637
Median (IQR)	92 (80, 116)	86 (77, 98)	

*2 cases and 4 controls had no late sample available.

^#^from fitting a conditional logistic regression model (continuous variables in log scale).

°Current use (i.e. use during the 3 months before sample) was an exclusion criterion.

§ available in 21 cases and 58 controls.

^ available in 24 cases and 56 controls.

### Biomarkers and risk of CVD

**Figure 1:**
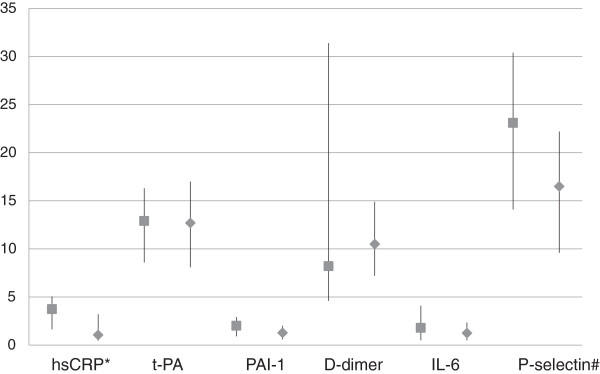
**Plasma levels of biomarkers on late samples in cases and matched controls.** Values indicate medians (full squares in cases and full diamonds in controls), bars indicate interquartile ranges. hsCRP = high-sensitivity C-reactive protein in mg/L; t-PA = tissue plasminogen activator in ng/mL; D-dimer in μg/100 mL; PAI-1 = plasminogen activator inhibitor-1 in μg/10 mL; IL-6 = interleukin-6 in pg/mL; P-selectin = platelet selectin in μg/100 mL. *p=0.002; #p=0.005 from fitting a conditional logistic regression (biomarkers in log scale) comparing cases and controls.

The 35 cases contributed 68 samples (35 early and 33 late samples) while the 74 controls contributed 145 samples (74 early and 71 late samples) for biomarkers analysis. In the unadjusted analysis comparing median values of biomarkers between cases and controls, the only biomarker consistently showing a higher median value in cases compared with controls was hsCRP, both in the analysis using the early samples (2.99 [IQR 1.13-5.99] mg/L in cases versus 1.62 [0.44-3.50] mg/L in controls, *P* = 0.09) and late samples (see Figure 1, *P* = 0.002). Higher median values were observed for t-PA in early samples (13.6 [11.1-17.0] ng/mL in cases versus 8.9 [6.3-13.2] ng/mL in controls, p<0.001) and P-selectin in late samples (Figure 1) in cases as compared to controls.

For hsCRP, in early samples, 6/35 (17% of cases) had levels in the lowest tertile compared with 26/74 (35% of controls). In contrast, the corresponding percentages with levels of hsCRP falling in the highest tertile were 14/35 (40%) and 26/74 (26%) for cases and controls, respectively. Similarly, for the late samples alone, 5/33 (15%) of cases and 23/71 (32%) of controls had hsCRP values in the lowest tertile vs. 14/33 (42%) and 14/71 (18%) in the highest tertile. For the combined data set, the corresponding values were 11/68 (16%) for cases and 49/145 (34%) for controls with hsCRP levels in the lowest tertile vs. 28/68 (41%) and 33/145 (23%) in the highest tertile.

Based on univariable and multivariable analyses, on late samples (Table 2) and the combined dataset (Table 3) a significantly increased risk of CVD events was observed for higher values of hsCRP, IL-6 and P-selectin, analyzed as categorical variables (Tables 2a and 2a), although a consistent association using the different sets of data was observed only for hsCRP and, less so, for P-selectin. Multivariable estimates were adjusted for all factors shown in the footnote of Tables 2 and 3. Similar results were obtained by analyzing biomarkers as log-transformed continuous variables (Tables 2b and 2b), with significant associations with hsCRP and P-selectin.

**Table 2:** Univariable and multivariable odds ratios for cardiovascular events according to biomarkers in late samples

***Biomarkers***	**Unadjusted OR (95% CI)**	**p-value**	Adjusted^* ^**OR (95% CI)**	**p-value**
	**(a)Biomarkers as categorical variables**			
**t-PA, ng/ml**				
<=8.7 (n=27)	1.00		1.00	
8.7-14.8 (n=26)	1.56 (0.48, 5.10)	0.459	2.54 (0.52, 12.31)	0.247
>14.8 (n=35)	0.99 (0.31, 3.14)	0.989	0.35 (0.05, 2.31)	0.278
Not measured (n=16)	2.99 (0.81, 10.95)	0.099	4.06 (0.50, 32.74)	0.188
**PAI-1, ng/ml**				
<=112 (n=36)	1.00		1.00	
112-207 (n=27)	1.15 (0.33, 3.96)	0.828	0.55 (0.11, 2.74)	0.468
>207 (n=29)	2.99 (0.98, 9.12)	0.054	3.20 (0.75, 13.76)	0.118
Not measured (n=12)	3.62 (0.91, 14.49)	0.069	3.02 (0.23, 39.02)	0.397
**D-dimer, ng/ml**				
<=67 (n=18)	1.00		1.00	
67-125 (n=27)	0.55 (0.15, 2.10)	0.384	0.32 (0.06, 1.84)	0.202
>125 (n=28)	0.72 (0.20, 2.61)	0.617	0.63 (0.12, 3.28)	0.585
Not measured (n=31)	1.70 (0.51, 5.62)	0.387	0.55 (0.10, 2.90)	0.482
**hsCRP, mg/L**				
< 0.9 (n=28)	1.00		1.00	
0.9-3.3 (n=31)	1.59 (0.46, 5.55)	0.467	5.25 (0.56, 49.67)	0.148
>3.3 (n=28)	4.28 (1.28, 14.36)	0.019	10.71 (1.03, 111.0)	0.047
Not measured (n=17)	2.54 (0.64, 10.11)	0.187	3.88 (0.23, 66.29)	0.349
**IL-6, pg/ml**				
< 0.3 (n=20)	1.00		1.00	
0.3-1.9 (n=34)	0.67 (0.20, 2.31)	0.529	0.13 (0.02, 0.86)	0.034
>1.9 (n=37)	1.39 (0.43, 4.46)	0.580	0.36 (0.06, 2.05)	0.249
Not measured (n=13)	2.03 (0.48, 8.50)	0.334	0.64 (0.04, 9.07)	0.738
**P-selectin, ng/ml**				
< 157 (n=38)	1.00		1.00	
157-236 (n=28)	1.23 (0.36, 4.19)	0.744	1.40 (0.29, 6.87)	0.676
>236 (n=25)	4.39 (1.43, 13.45)	0.010	3.94 (0.82, 18.88)	0.086
Not measured (n=13)	3.41 (0.88, 13.20)	0.075	2.84 (0.24, 33.51)	0.407
	**(b)Biomarkers as continuous variables (Log Scale)**			
**t-PA, ng/ml**				
Per log2 higher	0.97 (0.55, 1.74)	0.926	0.78 (0.35, 1.76)	0.554
**PAI-1, ng/ml**				
Per log2 higher	1.47 (0.96, 2.24)	0.073	1.40 (0.81, 2.42)	0.233
**D-dimer, ng/ml**				
Per log2 higher	0.89 (0.57, 1.41)	0.626	0.97 (0.55, 1.72)	0.918
**hsCRP, mg/L**				
Per log2 higher	1.61 (1.18, 2.18)	0.002	1.69 (1.09, 2.64)	0.020
IL-6, pg/ml				
Per log2 higher	1.09 (0.81, 1.45)	0.568	1.05 (0.72, 1.53)	0.799
**P-selectin, ng/ml**				
Per log2 higher	2.88 (1.38, 6.02)	0.005	3.14 (1.04, 9.45)	0.042

Estimate performed by a Conditional Logistic Regression Model; CI = confidence interval; HDL = high-density lipoprotein; hsCRP = high-sensitivity C-reactive protein; IL-6 = interleukin-6; IQR = interquartile range; OR = odds ratio; PAI-1 = plasminogen activator inhibitor-1; t-PA = tissue plasminogen activator. *Adjusted for duration of time between the date of samples and the analysis time, age, total cholesterol, HDL.

**Table 3:** Univariable and multivariable OR for CVD risk and biomarkers in early and late samples combined

***Biomarkers***	**Unadjusted OR (95% CI)**	***P *value**	Adjusted^* ^**OR (95% CI)**	***P *value**
	**(a)Biomarkers as categorical variables**			
**t-PA, ng/ml**				
<=8.7 (n=61)	1.00		1.00	
8.7-14.8 (n=60)	3.87 (1.61, 9.31)	0.003	1.58 (0.33, 7.65)	0.571
>14.8 (n=62)	3.23 (1.35, 7.76)	0.009	0.66 (0.12, 3.75)	0.642
Not measured (n=16)	3.92 (1.42, 10.84)	0.008	0.81 (0.09, 7.07)	0.853
**PAI-1, ng/ml**				
<=112 (n=63)	1.00		1.00	
112-207 (n=61)	1.03 (0.45, 2.32)	0.950	1.94 (0.37, 10.33)	0.435
>207 (n=65)	1.87 (0.86, 4.04)	0.113	2.15 (0.48, 9.63)	0.318
Not measured (n=12)	1.70 (0.62, 4.64)	0.300	0.52 (0.04, 7.73)	0.637
**D-dimer, ng/ml**				
<=67 (n=48)	1.00		1.00	
67-125 (n=48)	0.71 (0.30, 1.72)	0.451	0.49 (0.10, 2.27)	0.360
>125 (n=49)	0.71 (0.30, 1.72)	0.450	1.03 (0.18, 5.80)	0.972
Not measured (n=31)	1.27 (0.58, 2.76)	0.552	1.01 (0.21, 4.84)	0.987
**hsCRP, mg/L**				
< 0.9 (n=60)	1.00		1.00	
0.9-3.3 (n=62)	1.90 (0.81, 4.46)	0.142	5.70 (1.00, 32.51)	0.050
>3.3 (n=61)	3.63 (1.59, 8.29)	0.002	8.00 (1.23, 51.94)	0.029
Not measured (n=17)	2.29 (0.84, 6.28)	0.107	0.94 (0.06, 15.95)	0.968
**IL-6, pg/ml**				
< 0.3 (n=62)	1.00		1.00	
0.3-1.9 (n=62)	1.89 (0.81, 4.41)	0.143	2.69 (0.54, 13.40)	0.227
>1.9 (n=64)	3.94 (1.74, 8.90)	<.001	6.91 (1.21, 39.49)	0.030
Not measured (n=13)	2.69 (0.94, 7.67)	0.065	0.92 (0.06, 14.95)	0.954
**P-selectin, ng/ml**				
< 157 (n=62)	1.00		1.00	
157-236 (n=62)	0.95 (0.42, 2.13)	0.898	1.45 (0.29, 7.30)	0.649
>236 (n=64)	1.95 (0.91, 4.17)	0.085	6.20 (1.07, 35.89)	0.042
Not measured (n=13)	1.57 (0.58, 4.26)	0.378	0.66 (0.04, 10.09)	0.766
	**(b)Biomarkers as continuous variables (Log Scale)**			
**t-PA, ng/ml**				
per log_2_ higher	1.68 (1.09, 2.59)	0.019	1.15 (0.52, 2.56)	0.736
**PAI-1, ng/ml**				
per log_2_ higher	1.12 (0.84, 1.48)	0.449	1.20 (0.71, 2.04)	0.498
**D-dimer, ng/ml**				
per log_2_ higher	1.01 (0.79, 1.31)	0.911	0.89 (0.57, 1.38)	0.598
**hsCRP, mg/l**				
per log_2_ higher	1.37 (1.14, 1.66)	<.001	1.54 (1.03, 2.30)	0.036
**IL-6, pg/ml**				
per log_2_ higher	1.08 (0.90, 1.30)	0.408	1.22 (0.85, 1.74)	0.281
**P-selectin, ng/ml**				
per log_2_ higher	1.70 (1.05, 2.75)	0.032	3.35 (1.20, 9.38)	0.022

Conditional Logistic Regression Model; *CI* = confidence interval; HDL = high-density lipoprotein; hsCRP = high-sensitivity C-reactive protein; IL-6 = interleukin-6; IQR = interquartile range; NNRTI = nonnucleoside reverse transcriptase inhibitor; NRTI = nucleoside reverse transcriptase inhibitor; OR = odds ratio; PAI-1 = plasminogen activator inhibitor-1; PI = protease inhibitor; t-PA = tissue plasminogen activator.

^*^Adjusted for calendar year, duration of time between the date of samples and the analysis time, age, total cholesterol, HDL, CD4^+^ cell count and viral load, cumulative exposure to NRTI, NNRTI and PI prior to sample, and co-infection with hepatitis B or C.

None of the other considered biomarkers showed an independent association with the risk of CVD. Covariate adjustment showed that the magnitude of the association was underestimated in the unadjusted analysis as adjusted OR were even higher, especially for hsCRP. Use of the combined data set provided more precise estimates, as a result of increased statistical power (Tables 3a and b).

Sensitivity analyses using the clinically validated hsCRP categories [30] showed similar results. In the late samples data set, adjusted ORs for CVD events were 6.45 (95% CI, 0.64 to 64.42; *P* = 0.112) for values of 1–3 mg/L and 13.69 (95% CI, 1.35 to 138.9; *P* = 0.027) for values >3 mg/L, as compared to values of <1 mg/L. The corresponding values for the combined data set were 6.5 (95% CI, 1.0 to 41.7; *P* = 0.047) and 11.4 (95% CI, 1.8 to 72.3; *P* = 0.010).

We performed additional sensitivity analyses on the combined early and late samples dataset. In the multivariable models, a similar association of hsCRP levels with the risk of CVD events was observed after further adjusting for BMI (per log_2_ higher hsCRP, adjusted OR 1.45 (1.03, 2.04), p=0.032), for eGFR (per log_2_ higher hsCRP, adjusted OR 1.38 [95% CI 0.97-1.95], p=0.071) and for prior use of statins (per log_2_ higher hsCRP, adjusted OR 1.31 [95% CI 0.98-1.76], p=0.068).

In the main multivariable model including hsCRP as a continuous variable (log scale), and using the combined data set (Table 3b), the following other factors were independently associated with a greater risk of a CVD event: a higher total cholesterol (OR, 3.19 per 1 mmol/L higher [95% CI, 1.57 to 6.46]; *P* = 0.001), a lower HDL cholesterol (OR, 0.06 per 0.5 mmol/L higher [95% CI, 0.01 to 0.31]; *P* <0.001), a less recent calendar year of the stored sample (OR, 0.92 per 5 years more recent [95% CI, 0.90 to 0.94]; *P* <0.001) and shorter time between the date of sample and the analysis time (OR, 0.91 per month longer [95% CI, 0.86 to 0.96]; *P* <0.001).

## Discussion and conclusions

From the analysis using the combined set of early and late samples, we estimated that elevated hsCRP was associated with increased CVD risk in HIV-positive patients receiving cART (8-fold risk increase comparing patients with hsCRP levels >3.3 mg/L with those with <0.9 mg/L). A key finding was that the effect of hsCRP on CVD risk appeared to be independent of traditional CVD risk factors (total cholesterol and low HDL), as well as potential confounders, such as BMI, renal function and use of statins. Increased risk of a CVD event was also evident for higher values of IL-6 and P-selectin, although the association was weaker than for hsCRP. In addition, higher total cholesterol and lower HDL cholesterol were confirmed to be independent predictors of risk of CVD in our HIV-positive population.

The collection of early and late samples provided an opportunity to investigate whether the prognostic value of the studied biomarkers may differ according to specific objectives (i.e. to predict the short-term vs. the long-term risk of CVD). In the unadjusted analysis, median hsCRP levels were higher in cases (i.e. with CVD events) than controls (i.e. without CVD events) for both early and late samples, and more cases had hsCRP levels in the highest vs. lowest tertile, although this difference was more significant in late samples. Numerical problems due to sparse data in the analysis including only early samples, did not allow the calculation of adjusted estimates in this subset. Despite this limitation, our findings suggest that in HIV-infected patients hsCRP may be a useful marker to predict the medium-to-long term risk of experiencing CVD events (22 months after the date of measurement, on average), although the crude association of this marker with CVD events was actually stronger for the short term.

Several studies have investigated hsCRP levels in HIV-positive patients, and the association between hsCRP and CVD risk. HsCRP levels were not statistically different between untreated HIV-infected and uninfected individuals in one study [40], while in another, CRP levels were higher in HIV-positive patients than the general population [41]. Approximately 30% of patients with HIV receiving long-term cART were shown to have CRP levels >3.0 mg/L [41], the highest CRP levels being observed in those who were currently treated with ART [42]. Furthermore, elevated hsCRP levels have been observed in ART-treated compared with ART-naïve HIV-positive patients in another study [43].

In the analysis by Triant et al. including HIV-positive and -negative patients enrolled in a large US healthcare system, elevated CRP levels were associated with more than a four-fold increase in the risk of acute myocardial infarction compared with HIV-negative patients with normal CRP [44], but the study used either CRP or hsCRP tested by different assays, thus not allowing the analysis of specific thresholds. In a large international study, the baseline levels of CRP were independently associated with CVD events, including myocardial infarction, stroke, coronary revascularization, congestive heart failure, CVD death and peripheral artery disease [34] The present study substantiates their results in the Italian setting, using a standardized method for hsCRP determination, and identifies 3.3 mg/L as a potential threshold associated with an increased CVD risk in the HIV-infected population. Moreover, we were able to confirm the association between CRP and CVD risk after controlling for eGFR, a factor potentially associated with both inflammation and CVD risk. However, in disagreement with these findings, Ford et al. showed no association between hsCRP and CVD events in treated HIV-positive patients [2], while hsCRP was elevated in individuals who were at *low* risk of a CVD event in the study by Boger et al. [45].

Similar to hsCRP, IL-6 is a marker of inflammation and has been associated with coronary heart disease and endothelial dysfunction [46]: therefore its association with cardiovascular events in this study is not unexpected. An even stronger association between IL-6 and myocardial infarction has been observed in a previous large study [34]. Plasma P-selectin is almost exclusively secreted by platelets and is associated with platelet activation, blood cell endothelial adhesion and increased cardiovascular risk, in HIV negative patients [47].

The limitations of the current study include the inability to assess the risk of CVD associated with gender (only 9 females were included) and the matching variables (smoking status and diabetes). Also, we were unable to control our estimates for potential differences in systolic blood pressure because a measure of this parameter was available only in a minority of the patients studied (not shown). Furthermore, time from baseline was not one of the matching criteria as it is generally the case in case–control studies nested within a prospective cohort, although cases and controls were matched within the same cohort. In addition, controls have been, on average, exposed to cART for longer than cases (by inclusion criteria, controls had to have received cART for at least 5 years prior to the analysis time while there was no such restriction for cases), and, as a consequence, biomarkers have been measured in more recent calendar years. However, both factors were controlled for in the multivariable analysis. Another limitation relates to the different number of samples contributed by patients to the analysis, although there were only minimal imbalances. Finally, the fact that no association was found between t-PA, PAI-1 or D-dimer and CVD risk in this study does not exclude their role, as the study was not powered to detect such associations.

The importance of evaluating, preventing and managing CVD in patients with HIV is recognized in guidelines for the use of antiretroviral agents in HIV-infected adults [48]. Because hsCRP is cheap to measure and easily available it may become a clinically useful tool to monitor CVD risk in HIV-positive patients. Nevertheless, it has to be said that despite the fact that hsCRP was found to be associated with the risk of cardiovascular events independently of traditional risk factors in the HIV-uninfected population, the role of this biomarker in improving the prediction of CVD of traditional risk scores remains controversial even in the general population [49]. Moreover, several caveats apply: substantial intra-individual variation in hsCRP levels may represent a problem although, in the context of the general population, the predictive value of single measures of this marker was similar to that of repeated measures in the same subjects; evidence showing that lowering hsCRP reduces CVD events is available but not always consistent, calling into question whether hsCRP is a relevant therapeutic target; in addition, in a recent meta-analysis, CRP was associated with a range of disorders, and not just CVD [50].

In summary, our analysis shows that hsCRP is a candidate biomarker predicting CVD risk in HIV-infected patients receiving ART. Additional studies analyzing the influence of the addition of this marker on the prediction of cardiovascular risk scores are required before implementation of routine measuring for the scope of prediction into clinical practice.

## Appendix

Members of the ICONA Foundation Study

Governing body

M. Moroni (Chair), G Angarano, A Antinori, F Castelli, R Cauda, A d’Arminio Monforte, G Di Perri, M Galli, R Iardino, G Ippolito, A Lazzarin, CF Perno, O Armignacco, PL Viale, F Von Schlosser.

Scientific secretary

A d’Arminio Monforte

Steering committee

A Ammassari, M Andreoni, A Antinori, C Balotta, P Bonfanti, S Bonora, M Borderi, MR Capobianchi, A Castagna, F Ceccherini-Silberstein, P Cinque, A Cozzi-Lepri, A d’Arminio Monforte, A De Luca, M Gargiulo, C Gervasoni, E Girardi, A Gori, G Guaraldi, M Lichtner, S Lo Caputo, G Madeddu, F Maggiolo, G Marchetti, S Marcotullio, L Monno, R Murri, C Mussini, M Puoti, C Torti

Statistical and monitoring team

A Cozzi-Lepri, P Cicconi, I Fanti, T Formenti, L Galli, P Lorenzini

Participating physicians and centers

**Italy** A. Giacometti, A Costantini, A. Riva (Ancona); G. Angarano, L Monno, C Carrisa, (Bari); F. Maggiolo, G Lazzari (Bergamo); PL. Viale, M Borderi, G. Verucchi (Bologna); F Castelli, C. Torti, C. Minardi, (Brescia); T. Quirino, C Abeli (Busto Arsizio); P.E. Manconi, P. Piano (Cagliari); J Vecchiet, K Falasca (Chieti); L. Sighinolfi, D. Segala (Ferrara); F. Mazzotta, S. Lo Caputo (Firenze); G. Cassola, G Viscoli, A. Alessandrini, R. Piscopo, G Mazzarello (Genova); C. Mastroianni, V. Belvisi (Latina); P. Bonfanti, I. Caramma (Lecco); A. Chiodera, P. Castelli (Macerata); M Galli, A. Lazzarin, G. Rizzardini, M. Puoti, A. d’Arminio Monforte, AL Ridolfo, R Piolini, A Castagna, S Salpietro, A Galli, A Bigoloni, V Spagnuolo, L Carenzi, P Zucchi, M.C. Moioli, R Rossotti, P Cicconi, T Formenti (Milano); C. Mussini, L Bisio (Modena); A Gori, G Lapadula (Monza), N. Abrescia, A. Chirianni, MG Guida, M Gargiulo (Napoli); F Baldelli, B Belfiori (Perugia); G. Parruti, T Ursini (Pescara); G. Magnani, M.A. Ursitti (Reggio Emilia); R. Cauda, M Andreoni, A. Antinori, V Tozzi, V. Vullo, A. De Luca, A. d’Avino, M. Zaccarelli, L Gallo, E. Nicastro, R. Acinapura, M Capozzi, R Libertone, M. Lichtner, G Tebano, (Roma); M.S. Mura, G Madeddu (Sassari); P. Caramello, G. Di Perri, G.C. Orofino, M Sciandra (Torino); G. Pellizzer, V. Manfrin (Vicenza).

Ethics Committees of the Clinical Centers participating to the ICONA Foundation Study

Spedali Civili - P.zza Spedali Civili, 1 25125 – Brescia

Ospedale Amedeo di Savoia - C.so Svizzera, 164–10149 Torino

Ospedale A Manzoni - Via dell’Eremo 9/11 - 22053 Lecco

Ospedale Busto Arsizio - Piazzale Solaro, 3–21052 Busto Arsizio (VA)

Policlinico Sant’Orsola Università di Bologna - Via Massarenti, 11–40138 Bologna

Arcispedale S Maria Nuova - V.le Risorgimento, 80 42100 Reggio Emilia

Azienda Policlinico di Modena -Via del Pozzo, 71–41100 – Modena

Azienda Ospedaliero Universitaria di Ferrara Via Aldo Moro, 8 44124 Cona - Ferrara

Università Politecnica Marche - Ospedale Umberto I L. go Cappelli, 1–60121 Ancona

Azienda Ospedaliera Universitaria Osp Riuniti di Ancona - 60020 - Torrette di Ancona

Presidio Ospedaliero AUSL 9 - Via Santa Lucia - 62100 Macerata

Istituto Nazionale per le Malattie Infettive IRCCS “L Spallanzani ”-Via Portuense, 292–00149 Roma

Università di Perugia Policlinico Monteluce - Via A. Brunamonti - 06122 Perugia

Policlinico Umberto I, Univ La Sapienza - V.le del Policlinico, 155–00163 Roma

Fondazione PTV - Policlinico Tor Vergata - Viale Oxford, 81–00133 Roma

Università “Aldo Moro” - P. le G. Cesare, 11–70124 Bari

Azienda Ospedaliera Cotugno - Via G. Quagliarello - 80131 Napoli

Università “G D’Annunzio” - Via Valignani - 66100 Chieti

USL di Pescara VIA FONTE ROMANA, N ° 8–65124 PESCARA

## Competing interests

ADL received speaker honoraria and fees for attending advisory boards from ViiV Healthcare, Gilead, Abbott Virology, Janssen and Siemens Diagnostics. MC has been a paid consultant for Merck Sharp & Dohme, Italy and has been employed by Bristol-Myers-Squibb, Italy since May 10^th^, 2010 to Feb 28^th^ 2011. ADM received speaker honoraria and fees for attending advisory boards from GlaxoSmithKline, Gilead, Bristol-Myers-Squibb, Boehringer-Ingelheim, Abbott Virology, Tibotec.

No other member of the Icona Foundation Study has any financial or personal relationships with people or organizations that could inappropriately influence this work, although most members of the group have, at some stage in the past, received funding from a variety of pharmaceutical companies for research, travel grants, speaking engagements or consultancy fees.

## Authors’ contributions

Conception and design: ADL, KGD, MC, Ad’AM. Analysis and interpretation of the data: AC-L, ADL, LI, ADC, Ad’AM. Critical review and revision of the article for scientific accuracy and intellectual content: all. Final approval of the article: all. Statistical expertise: AC-L. Administrative, technical or logistic support: MC, RC, Ad’AM. Collection and assembly of data: ADC, MC, KGD, MP, AC-L, Ad’AM, AG, FG, GS.

## Pre-publication history

The pre-publication history for this paper can be accessed here:

http://www.biomedcentral.com/1471-2334/13/414/prepub

## References

[B1] Palella FJ Jr Delaney KM Moorman AC Loveless MO Fuhrer J Satten GA Aschman DJ Holmberg SD Declining morbidity and mortality among patients with advanced human immunodeficiency virus infection. HIV Outpatient Study Investigators N Engl J Med 1998 338 853 860 10.1056/NEJM199803263381301 9516219

[B2] Ford ES Greenwald JH Richterman AG Rupert A Dutcher L Badralmaa Y Natarajan V Rehm C Hadigan C Sereti I Traditional risk factors and D-dimer predict incident cardiovascular disease events in chronic HIV infection AIDS 2010 24 1509 1517 10.1097/QAD.0b013e32833ad914 20505494 PMC2884071

[B3] Currier JS Lundgren JD Carr A Klein D Sabin CA Sax PE Schouten JT Smieja M Working Group 2 Epidemiological evidence for cardiovascular disease in HIV-infected patients and relationship to highly active antiretroviral therapy Circulation 2008 118 e29 e35 10.1161/CIRCULATIONAHA.107.189624 18566319 PMC5153327

[B4] Triant VA Lee H Hadigan C Grinspoon SK Increased acute myocardial infarction rates and cardiovascular risk factors among patients with human immunodeficiency virus disease J Clin Endocrinol Metab 2007 92 2506 2512 10.1210/jc.2006-2190 17456578 PMC2763385

[B5] Law MG Friis-Møller N El-Sadr WM Weber R Reiss P D’Arminio Monforte A Thiébaut R Morfeldt L De Wit S Pradier C Calvo G Kirk O Sabin CA Phillips AN Lundgren JD D:A:D Study Group The use of the Framingham equation to predict myocardial infarctions in HIV-infected patients: comparison with observed events in the D:A:D Study HIV Med 2006 7 218 230 10.1111/j.1468-1293.2006.00362.x 16630034

[B6] Maggi P Quirino T Ricci E De Socio GV Gadaleta A Ingrassia F Perilli F Lillo A Bonfanti P Cardiovascular risk assessment in antiretroviral-naïve HIV patients AIDS Patient Care STDS 2009 23 809 813 10.1089/apc.2009.0102 19824809

[B7] Teitel JM Shore A Read SE Schiavone A Immune function of vascular endothelial cells is impaired by HIV J Infect Dis 1989 160 551 552 10.1093/infdis/160.3.551 2788201

[B8] Zietz C Hotz B Stürzl M Rauch E Penning R Löhrs U Aortic endothelium in HIV-1 infection: chronic injury, activation, and increased leukocyte adherence Am J Pathol 1996 149 1887 1898 8952525 PMC1865334

[B9] de Gaetano DK Rabagliati R Iacoviello L Cauda R HIV infection, HAART, and endothelial adhesion molecules: current perspectives Lancet Infect Dis 2004 4 213 222 10.1016/S1473-3099(04)00971-5 15050939

[B10] de Gaetano DK Rabagliati R Tumbarello M Tacconelli E Amore C Cauda R Iacoviello L Increased soluble markers of endothelial dysfunction in HIV-positive patients under highly active antiretroviral therapy AIDS 2003 17 765 768 10.1097/00002030-200303280-00020 12646805

[B11] Currier JS Taylor A Boyd F Dezii CM Kawabata H Burtcel B Maa JF Hodder S Coronary heart disease in HIV-infected individuals J Acquir Immune Defic Syndr 2003 33 506 512 10.1097/00126334-200308010-00012 12869840

[B12] Bergersen BM Cardiovascular risk in patients with HIV Infection: impact of antiretroviral therapy Drugs 2006 66 1971 1987 10.2165/00003495-200666150-00006 17100407

[B13] Wolf K Tsakiris DA Weber R Erb P Battegay M Swiss HIV Cohort Study Antiretroviral therapy reduces markers of endothelial and coagulation activation in patients infected with human immunodeficiency virus type 1 J Infect Dis 2002 185 456 462 10.1086/338572 11865397

[B14] Torriani FJ Komarow L Parker RA Cotter BR Currier JS Dubé MP Fichtenbaum CJ Gerschenson M Mitchell CK Murphy RL Squires K Stein JH ACTG 5152s Study Team Endothelial function in human immunodeficiency virus-infected antiretroviral-naive subjects before and after starting potent antiretroviral therapy: The ACTG (AIDS Clinical Trials Group) Study 5152s J Am Coll Cardiol 2008 52 569 576 10.1016/j.jacc.2008.04.049 18687253 PMC2603599

[B15] Worm SW Sabin C Weber R Reiss P El-Sadr W Dabis F De Wit S Law M Monforte AD Friis-Møller N Kirk O Fontas E Weller I Phillips A Lundgren J Risk of myocardial infarction in patients with HIV infection exposed to specific individual antiretroviral drugs from the 3 major drug classes: the data collection on adverse events of anti-HIV drugs (D:A:D) study J Infect Dis 2010 201 318 330 10.1086/649897 20039804

[B16] Lang S Mary-Krause M Cotte L Gilquin J Partisani M Simon A Boccara F Costagliola D Clinical Epidemiology Group of the French Hospital Database on HIV Impact of individual antiretroviral drugs on the risk of myocardial infarction in human immunodeficiency virus-infected patients: a case–control study nested within the French Hospital Database on HIV ANRS cohort CO4 Arch Intern Med 2010 170 1228 1238 10.1001/archinternmed.2010.197 20660842

[B17] Friis-Møller N Weber R Reiss P Thiébaut R Kirk O D’Arminio Monforte A Pradier C Morfeldt L Mateu S Law M El-Sadr W De Wit S Sabin CA Phillips AN Lundgren JD DAD study group Cardiovascular disease risk factors in HIV patients--association with antiretroviral therapy. Results from the DAD study AIDS 2003 17 1179 1193 10.1097/00002030-200305230-00010 12819520

[B18] Stein JH Klein MA Bellehumeur JL McBride PE Wiebe DA Otvos JD Sosman JM Use of human immunodeficiency virus-1 protease inhibitors is associated with atherogenic lipoprotein changes and endothelial dysfunction Circulation 2001 104 257 262 10.1161/01.CIR.104.3.257 11457741

[B19] Holmberg SD Moorman AC Williamson JM Tong TC Ward DJ Wood KC Greenberg AE Janssen RS HIV Outpatient Study (HOPS) investigators. Protease inhibitors and cardiovascular outcomes in patients with HIV-1 Lancet 2002 360 1747 1748 10.1016/S0140-6736(02)11672-2 12480430

[B20] Friis-Møller N Reiss P Sabin CA Weber R Monforte A El-Sadr W Thiébaut R De Wit S Kirk O Fontas E Law MG Phillips A Lundgren JD DAD Study Group Class of antiretroviral drugs and the risk of myocardial infarction N Engl J Med 2007 356 1723 1735 17460226 10.1056/NEJMoa062744

[B21] Wilson SL Scullard G Fidler SJ Weber JN Poulter NR Effects of HIV status and antiretroviral therapy on blood pressure HIV Med 2009 10 388 394 10.1111/j.1468-1293.2009.00699.x 19490176

[B22] Sabin CA Worm SW Weber R Reiss P El-Sadr W Dabis F De Wit S Law M D’Arminio Monforte A Friis-Møller N Kirk O Pradier C Weller I Phillips AN Lundgren JD D:A:D Study Group Use of nucleoside reverse transcriptase inhibitors and risk of myocardial infarction in HIV-infected patients enrolled in the D:A:D study: a multi-cohort collaboration Lancet 2008 371 1417 1426 18387667 10.1016/S0140-6736(08)60423-7 PMC2688660

[B23] Behrens GM Reiss P Abacavir and cardiovascular risk Curr Opin Infect Dis 2010 23 9 14 10.1097/QCO.0b013e328334fe84 19996748

[B24] Ridker PM Clinical application of C-reactive protein for cardiovascular disease detection and prevention Circulation 2003 107 363 369 10.1161/01.CIR.0000053730.47739.3C 12551853

[B25] Ridker PM Cannon CP Morrow D Rifai N Rose LM McCabe CH Pfeffer MA Braunwald E Pravastatin or Atorvastatin Evaluation and Infection Therapy-Thrombolysis in Myocardial Infarction 22 (PROVE IT-TIMI 22) Investigators C-reactive protein levels and outcomes after statin therapy N Engl J Med 2005 352 20 28 10.1056/NEJMoa042378 15635109

[B26] McMurray JJ Kjekshus J Gullestad L Dunselman P Hjalmarson A Wedel H Lindberg M Waagstein F Grande P Hradec J Kamenský G Korewicki J Kuusi T Mach F Ranjith N Wikstrand J CORONA Study Group Effects of statin therapy according to plasma high-sensitivity C-reactive protein concentration in the Controlled Rosuvastatin Multinational Trial in Heart Failure (CORONA): aretrospective analysis Circulation 2009 120 2188 2196 10.1161/CIRCULATIONAHA.109.849117 19917888

[B27] Lowe GD Fibrin D-dimer and cardiovascular risk Semin Vasc Med 2005 5 387 398 10.1055/s-2005-922485 16302161

[B28] Asselbergs FW Williams SM Hebert PR Coffey CS Hillege HL Navis G Vaughan DE van Gilst WH Moore JH Gender-specific correlations of plasminogen activator inhibitor-1 and tissue plasminogen activator levels with cardiovascular disease-related traits J Thromb Haemost 2007 5 313 320 10.1111/j.1538-7836.2007.02311.x 17092303

[B29] Zakynthinos E Pappa N Inflammatory biomarkers in coronary artery disease J Cardiol 2009 53 3 317 333 10.1016/j.jjcc.2008.12.007 19477372

[B30] Pearson TA Mensah GA Alexander RW Anderson JL Cannon RO 3rd Criqui M Fadl YY Fortmann SP Hong Y Myers GL Rifai N Smith SC Jr Taubert K Tracy RP Vinicor F Centers for Disease Control and Prevention Markers of inflammation and cardiovascular disease: application to clinical and public health practice: a statement for healthcare professionals from the Centers for Disease Control and Prevention and the American Heart Association Circulation 2003 107 499 511 10.1161/01.CIR.0000052939.59093.45 12551878

[B31] Duprez DA Kuller LH Tracy R Otvos J Cooper DA Hoy J Neuhaus J Paton NI Friis-Moller N Lampe F Liappis AP Neaton JD INSIGHT SMART Study Group Lipoprotein particle subclasses, cardiovascular disease and HIV infection Atherosclerosis 2009 207 524 529 10.1016/j.atherosclerosis.2009.05.001 19515371 PMC2818719

[B32] Mastroianni CM Lichtner M Mengoni F D’Agostino C d’Ettorre G Forcina G Santopadre P Massetti AP Vullo V Changes in circulating levels of soluble cell adhesion molecules following highly active antiretroviral treatment of HIV-1-infected patients Clin Immunol 2000 95 212 217 10.1006/clim.2000.4865 10866128

[B33] Tungsiripat M Adell J McComsey GA Relationship between inflammatory markers, endothelial activation markers, and carotid intima-media thickness in HIV-infected patients receiving antiretroviral therapy Clin Infect Dis 2009 49 1119 1127 10.1086/605578 19712036 PMC3895473

[B34] Duprez DA Neuhaus J Kuller LH Inflammation, coagulation and cardiovascular disease in HIV-infected individuals PLoS One 2012 7 e44454 10.1371/journal.pone.0044454 22970224 PMC3438173

[B35] Di Giambenedetto S Bracciale L Colafigli M Cattani P Pinnetti C Bacarelli A Prosperi M Fadda G Cauda R De Luca A Declining prevalence of HIV-1 drug resistance in treatment-failing patients: a clinical cohort study Antivir Ther 2007 12 835 839 17713168

[B36] d’Arminio Monforte A Lepri AC Rezza G Pezzotti P Antinori A Phillips AN Angarano G Colangeli V De Luca A Ippolito G Caggese L Soscia F Filice G Gritti F Narciso P Tirelli U Moroni M Insights into the reasons for discontinuation of the first highly active antiretroviral therapy (HAART) regimen in a cohort of antiretroviral naïve patients. I.CO.N.A. Study Group. Italian Cohort of Antiretroviral-Naïve Patients AIDS 2000 14 499 507 10.1097/00002030-200003310-00005 10780712

[B37] Sasieni P A note on the presentation of matched case–control data Stat Med 1992 11 617 620 10.1002/sim.4780110506 1594804

[B38] Greenland S Schwartzbaum JA Finkle WD Problems due to small samples and sparse data in conditional logistic regression analysis Am J Epidemiol 2000 151 531 539 10.1093/oxfordjournals.aje.a010240 10707923

[B39] Zeger SL Liang KY Longitudinal data analysis for discrete and continuous outcomes Biometrics 1986 42 121 130 10.2307/2531248 3719049

[B40] Baker J Ayenew W Quick H Hullsiek KH Tracy R Henry K Duprez D Neaton JD High-density lipoprotein particles and markers of inflammation and thrombotic activity in patients with untreated HIV infection J Infect Dis 2010 201 285 292 10.1086/649560 19954384 PMC2798007

[B41] Noursadeghi M Miller RF Clinical value of C-reactive protein measurements in HIV-positive patients Int J STD AIDS 2005 16 438 441 15969780 10.1258/0956462054094006

[B42] Masiá M Bernal E Padilla S Graells ML Jarrín I Almenar MV Molina J Hernández I Gutiérrez F The role of C-reactive protein as a marker for cardiovascular risk associated with antiretroviral therapy in HIV-infected patients Atherosclerosis 2007 195 1 167 171 10.1016/j.atherosclerosis.2006.09.013 17049532

[B43] Guimarães MM Greco DB Figueiredo SM Fóscolo RB Oliveira AR Jr Machado LJ High-sensitivity C-reactive protein levels in HIV-infected patients treated or not with antiretroviral drugs and their correlation with factors related to cardiovascular risk and HIV infection Atherosclerosis 2008 201 2 434 439 10.1016/j.atherosclerosis.2008.02.003 18359028

[B44] Triant VA Meigs JB Grinspoon SK Association of C-reactive protein and HIV infection with acute myocardial infarction J Acquir Immune Defic Syndr 2009 51 268 273 10.1097/QAI.0b013e3181a9992c 19387353 PMC2763381

[B45] Boger MS Shintani A Redhage LA Mitchell V Haas DW Morrow JD Hulgan T Highly sensitive C-reactive protein, body mass index, and serum lipids in HIV-infected persons receiving antiretroviral therapy: a longitudinal study J Acquir Immune Defic Syndr 2009 52 4 480 487 10.1097/QAI.0b013e3181b939e5 19911471 PMC2794651

[B46] Erzen B Sabovic M Sebestjen M Keber I Poredos P Interleukin-6 correlates with endothelial dysfunction in young post-myocardial infarction patients Cardiology 2007 107 2 111 116 10.1159/000094588 16864964

[B47] Blann AD Nadar SK Lip GY The adhesion molecule P-selectin and cardiovascular disease Eur Heart J 2003 24 2166 2179 10.1016/j.ehj.2003.08.021 14659768

[B48] Panel on Antiretroviral Guidelines for Adults and Adolescents Guidelines for the use of antiretroviral agents in HIV-1-infected adults and adolescents. Department of Health and Human Services Available at http://aidsinfo.nih.gov/ContentFiles/AdultandAdolescentGL.pdf. Accessed January 11th 2013. Last updated March 27th 2012

[B49] Ben-Yehuda O High-sensitivity C-reactive protein in every chart? The use of biomarkers in individual patients J Am Coll Cardiol 2007 49 2139 2141 10.1016/j.jacc.2007.04.007 17531664

[B50] Kaptoge S Di Angelantonio E Lowe G Pepys MB Thompson SG Collins R Danesh J Collaboration Emerging Risk Factors C-reactive protein concentration and risk of coronary heart disease, stroke, and mortality: an individual participant meta-analysis Lancet 2010 375 9709 132 140 20031199 10.1016/S0140-6736(09)61717-7 PMC3162187

